# Performance of cell-free DNA sequencing-based non-invasive prenatal testing: experience on 36,456 singleton and multiple pregnancies

**DOI:** 10.1186/s12920-021-00941-y

**Published:** 2021-03-30

**Authors:** Marco La Verde, Luigia De Falco, Annalaura Torella, Giovanni Savarese, Pasquale Savarese, Raffaella Ruggiero, Anna Conte, Vera Fico, Marco Torella, Antonio Fico

**Affiliations:** 1 Department of Woman, Child and General and Specialized Surgery, Obstetrics and Gynecology Unit, University of Campania “Luigi Vanvitelli”, Naples, Italy; 2 AMES, Centro Polidiagnostico Strumentale, Srl, Naples, Italy; 3 Department of Precision Medicine, University of Campania “Luigi Vanvitelli”, Naples, Italy

**Keywords:** Whole-genome sequencing, Non-invasive prenatal testing, NIPT, Cell-free DNA, Screening, Aneuploidy, Trisomy, Down syndrome, Chromosome abnormality, VeriSeq NIPT solution

## Abstract

**Background:**

This paper describes the clinical practice and performance of cell-free DNA sequencing-based non-invasive prenatal testing (NIPT) as a screening method for fetal trisomy 21, 18, and 13 (T21, T18, and T13) and sex chromosome aneuploidies (SCA) in a general Italian pregnancy population.

**Methods:**

The AMES-accredited laboratory offers NIPT in maternal blood as a screening test for fetal T21, T18, T13 and SCA. Samples were sequenced on a NextSeq 550 (Illumina) using the VeriSeq NIPT Solution v1 assay.

**Results:**

A retrospective analysis was performed on 36,456 consecutive maternal blood samples, including 35,650 singleton pregnancies, 800 twin pregnancies, and 6 triplet pregnancies. Samples were tested between April 2017 and September 2019. The cohort included 46% elevated-risk and 54% low-risk patients. A result indicative of a classic trisomy was found in 356 (1%) of singleton or twin samples: 254 T21, 69 T18, and 33 T13. In addition, 145 results (0.4%) were indicative of a SCA. Of the combined 501 screen-positive cases, 484 had confirmatory diagnostic testing. NIPT results were confirmed in 99.2% (247/249) of T21 cases, 91.2% (62/68) of T18 cases, 84.4% (27/32) of T13 cases, and 86.7% (117/135) of SCA cases. In the 35,955 cases reported as unaffected by a classic trisomy or SCA, no false negative cases were reported. Assuming that false negative results would be reported, the sensitivity of NIPT was 100.00% for T21 (95% Cl 98.47–100.0), T18 (95% Cl 94.17–100.0), and T13 (95% Cl 87.54–100.0). The specificities were 99.99% (95% Cl 99.98–100.0), 99.98% (95% Cl 99.96–100.0), 99.99% (95% Cl 99.97–100.0), and 99.95% (95% Cl 99.92–99.97) for T21, T18, T13, and SCA, respectively.

**Conclusion:**

This retrospective analysis of a large cohort of consecutive patients who had whole-genome sequencing-based NIPT for classic trisomies and SCA shows excellent detection rates and low false positive rates.

**Supplementary Information:**

The online version contains supplementary material available at 10.1186/s12920-021-00941-y.

## Background

During the last few decades, prenatal screening and diagnosis of fetal chromosome alterations and ultrasound detection of structural anomalies have rapidly evolved [1]. “Traditional” prenatal screening methods for chromosome anomalies combining maternal age, ultrasound markers, and maternal serum markers, reach 80%–90% sensitivity and present a 5% false positive (FP) rate [1, 2]. A definitive diagnosis of the fetal karyotype needs confirmatory invasive testing, either first-trimester chorionic villus sampling (CVS) or second-trimester amniocentesis. Both entail a miscarriage risk of 0.1–0.5% [3]. The presence of circulating cell-free DNA (cfDNA) from the placenta in the maternal circulation was first demonstrated by Lo et al. [4]. This finding, combined with the discovery of massively parallel sequencing (MPS) technologies [5, 6], made it possible to develop a highly accurate non-invasive prenatal test (NIPT) for fetal aneuploidy detection, with a highly improved positive predictive value (PPV). This has allowed for a reduction in the number of invasive procedures and associated risks [7], as well as a reduction in the number of patients exposed to anxiety resulting from abnormal screening results [8]. Since its commercial launch in 2011, cfDNA-based non-invasive prenatal testing (NIPT) has permitted screening for T21, T18, and T13 with high specificity and sensitivity in both high-and low-risk populations [9, 10]. In addition, singleton pregnancies allow the identification of fetal sex chromosome aneuploidies (SCAs), although the occurrence of maternal mosaics and lack of visible features at birth complicate assessment of test performance [11, 12]. Large-scale studies and meta-analyses show good performance across technologies and populations [10, 11, 13, 14]. As data on twin pregnancies are limited [15], the use of NIPT in twin and higher-order multiple pregnancies has been recommended with caution [16–18].

Here, we report on a large, single-centre cohort of consecutive singleton and multiple gestation patients tested for classic trisomies with a whole-genome sequencing (WGS)-based NIPT. Follow-up invasive testing was available for 96.6% of screen-positive cases. The results show that WGS-based NIPT performs equally well in twin and singleton pregnancies.

## Methods

### Study population and study design

The study included a retrospective investigation of 36,456 pregnant women referred to the AMES laboratory in Naples for NIPT between April 2017 and September 2019. The laboratory is accredited (UNI EN ISO 9001:2008) for prenatal testing and genetic disease testing. The study population included both low-risk women choosing NIPT as a first-tier test and women considered to be at elevated risk for fetal aneuploidies based on maternal age ≥ 35 years, a previous pregnancy with aneuploidy, first trimester combined test results (risk above 1/270 or 1/300, depending on individual hospital criteria), abnormal ultrasound findings (including a nuchal translucency of > 3.5 mm), parents with balanced chromosomal abnormalities or other chromosomal rearrangements of sufficient size to be detectable by our assay, or a family history of aneuploidy.

The cohort included singleton, monochorionic and dichorionic twin pregnancies and triplet pregnancies both naturally conceived or with assisted reproductive techniques (ART). Chorionicity was assessed by ultrasound. Patients were accepted from a gestational age of 9 weeks onwards. Participants below 18 years of age were excluded. All patients received pre-test counselling, and written informed consent was obtained before blood collection. In the case of an abnormal test result, additional counselling was provided by a clinical geneticist or obstetrician [19], and confirmatory testing in material obtained via amniocentesis or chorionic villus sampling was offered. The study was approved by the local Ethics Committee of University of Campania “Luigi Vanvitelli”, Naples, Italy. No other administrative permissions or licenses were required to access the clinical/personal patient data.

### Sample collection and processing

A blood sample was either collected at the AMES Laboratory (60% of samples) or sent to the laboratory from within Italy (40%). The latter samples were sent at a controlled temperature of 4 °C, and the maximum interval between blood draw and arrival at AMES was 5 days. CfDNA was extracted from 900 μL of maternal plasma according to the VeriSeq™ NIPT Solution v1 instructions for use [20]. Briefly, cfDNA extraction and purification were achieved by adsorption onto a binding plate, the binding plate was washed to remove contaminants, followed by eluting. The pipeline included an automated library preparation (VeriSeq NIPT Solution v1, Microlab STAR, Illumina) followed by WGS sequencing on a NextSeq 550 (Illumina). VeriSeqNIPT Assay Software v1 (www.illumina.com/NIPTsoftware) was used for the analysis of the aneuploidy status and fetal fraction. The generated WGS data were streamed to the VeriSeq NIPT Analysis Server, where the software filtered and aligned the WGS reads to a human reference genome. The software uses a counting‐based algorithm to generate the log-likelihood ratio (LLR) scores for chromosomes 13, 18, and 21, as well as NCV_X and NCV_Y scores for sex classification for each sample. LLR thresholds for calling a sample high or low risk were internally validated, and a decision tree was agreed upon for handling failures [21]. Samples failed when the sequencing coverage was judged insufficient based on the fetal fraction estimate for the sample, as indicated by the Individualized Fetal Fraction Confidence Test (iFACT), which is a quality control parameter of the VeriSeq^TM^ NIPT Solution v1. If samples repeatedly failed due to data outside of the expected range (DOER), a genome-wide data analysis was performed with an in-house developed algorithm to identify whether rare aneuploidy was the cause of repeated failure. Resampling and reanalysis were performed at no extra cost. Singleton and multiple gestation pregnancy samples were handled in the same way. The statistical analysis of the data was conducted utilizing the statistical set SPSS for Windows (version 20 SPSS Inc., Chicago, IL).

### Report delivery and clinical follow-up/patient management

NIPT results were delivered and explained within 1–2 working days of receiving the blood sample in the laboratory. In the case of high-risk results, personal post-test counselling was performed by a clinical geneticist, and patients were recommended to have confirmatory invasive prenatal testing. The recommendation for the type of follow-up test (amniocentesis [AC] or chorionic villus sampling [CVS]) was based on the type of chromosomal anomaly, gestational age, and patient preference [22]. Postnatal cytogenetic confirmation included confirmation in cases of fetal demise or miscarriage. When a maternal origin of the chromosomal aberration was suspected, the initial recommendation was maternal testing.

### Collection of follow-up and pregnancy outcome data

Follow-up diagnostic testing in cases of high-risk NIPT results was performed in the AMES laboratories at no additional cost. In case of low-risk NIPT results, data on pregnancy outcomes were provided by the treating physician or by the patient. Follow-up data were entered in a database.

### Data statement

Protocols and deidentified, aggregated data that underlie the results reported in this article are available for non-commercial scientific purposes upon reasonable request from the corresponding author.

## Results

### Participants

Between 18 April 2017 and 30 September 2019, 36,456 blood samples were sent for NIPT. The patient and pregnancy characteristics are shown in Table 1. The cohort included 46% elevated-risk and 54% low-risk patients.The mean gestational age at the time of blood draw was 12 weeks and 2 days (range 9^1/7^–32^6/7^). In 76.2% of the patients, blood was drawn in the first trimester of pregnancy. Maternal age ranged from 18 to 48 years, with a mean of 35.4 years. The average fetal DNA fraction was 9.54% ± 3.72%. Among all pregnancies, 5% (1807/36,456) were conceived using assisted reproductive technologies (ART) and 0.04% (16/36,456) were conceived through egg donation. The latter were excluded from the average maternal age calculations.

**Table 1:** Demographic characteristics of women requesting cell-free DNA (cfDNA) screening for common trisomies and sex chromosome aneuploidies

Characteristics	n (%) unless otherwise stated
Low risk NIPT results	35,955 (98.6)
Singleton pregnancies (%)	35,650 (97.8)
Twin pregnancies (%)	800 (2.2)
Triplet pregnancies,(%)	6 (0.02)
ART Pregnancies(%)	1807 (5)
Twin pregnancies (%)	403 (22.3)
Egg donation (%)	16 (0.9)
Maternal age (yrs) mean (range)*	35.4 (18–48)
< 35(%)	19,693 (54)
≥ 35(%)	16,763 (46)
Gestational age (wks + days) mean (range)	12 + 2 (9 + 1–32 + 6)
Samples drawn in first trimester (< 13 + 6 wks)	27,780 (76.2%)
Fetal fraction mean ± SD	9.55 ± 3.72
Fetal fraction first trimester mean ± SD	9.53 ± 3.69

*ART* artificial reproductive technology

*We excluded pregnancies with egg donation

Among the cohort of 36,456, 2.2% (800) were twin pregnancies and 0.02% (6) were triplet pregnancies. Of the 800 twin pregnancies, 718 (89.7%) were dichorionic and 82 (10.3%) were monochorionic. The mean maternal age among twin pregnancies was 38.2 (range 34.3–45.0) years, and therefore slightly higher than that in the whole cohort. The mean gestational age at sampling in twin pregnancies was similar to that in the whole cohort: 12 weeks and 3 days (range 10^1/7^–19^6/7^). Approximately 50% (403/800) of twin cases were conceived using assisted reproductive techniques.

Overall, 35,955 (98.6%) patients received a low-risk NIPT result, and 501 (1.4%) received a high-risk NIPT result (including SCA). The high-risk results were 254 T21, 69 T18, 33 T13 and 145 SCA. Thus, SCA comprised 28.9% of the abnormal results. Elevated risk results for classic trisomies were found in 1.5% (12/806) of twin pregnancies and 1% (344/35,650) of singleton pregnancies.

### Test results and pregnancy outcome data (Tables 2 and 3)

**Table 2:** NIPT performance for detecting trisomies 21, 18, and 13 and sex chromosome aneuploidies: cases with high risk NIPT results, including singleton and twin pregnancies

Nipt results	n	Loss to follow-up	No confirmatory testing		Confirmatory diagnostic testing		
			IUFD (n)	TOP (n)	TP (n)	FP (n)	PPV% (95% CI)
T21	254	1	4	0	247	2	99.2 (99.1, 99.3)
T18	69		0	1	62	6	91.2 (91.0, 91.6)
T13	33		1	0	27	5	84.4 (84.5, 85.2)
SCA	145		8	2	117	18	86.7 (86.2, 87.9)
Total	501	1	13	3	453	31	93.6 (91.4, 95.8)

*CI* confidence interval, *IUFD* intrauterine fetal demise, *n* number, *NIPT* non-invasive prenatal test, *PPV* positive predictive value, *SCA* sex chromosome aneuploidy, *TOP* termination of pregnancy, *T* trisomy, *TP* true-positive, *FP* false positive

**Table 3:** NIPT performance for detecting trisomies 21, 18, 13 and sex chromosome aneuploidies: all cases

n = 36,000	TP	FP	TN	Reported FN	Sensitivity	Specificity	PPV	NPV
					TP/(TP + FN)	TN/(TN + FP)	TP/(TP + FP)	TN/(TN + FN)
					% (95% Cl)	% (95% Cl)	% (95% Cl)	% (95% Cl)
T21	247	2	35,410	0	100 247/247	99.99 35,410/35,412	99.20 247/249	100 35,410/35,410
					(98.47, 100.0)	(99.98, 100.0)	(98.10, 99.30)	(99.99, 100.0)
T18	62	6	35,591	0	100 62/62	99.98 35,591/35,597	91.20 62/68	100 35,591/35,591
					(94.17, 100.0)	(99.96, 100.0)	(84.54, 97.86)	(99.99, 100.0)
T13	27	5	35,627	0	100 27/27	99.99 35,627/35,632	84.40 27/32	100 35,627/35,627
					(87.54, 100.0)	(99.97, 100.0)	(83.15, 96.90)	(99.99, 100.0)
SCA	117	18	35,524	0	100 117/117	99.95 35,524/35,542	86.7 117/135	100 35,524/35,524
					(96.82, 100.0)	(99.92, 99.97)	(81.0, 92.38)	(99.99, 100.0)
All	453	31	35,175	0	100 453/453	99.91 35,175/35,206	93.6 453/484	100 35,175/35,175
					(99.19, 100.0)	(99.88, 99.94)	(91.44, 95.76)	(99.99, 100.0)

*CI* confidence interval, *T21* trisomy 21, *T18* trisomy 18, *T13* trisomy 13, *SCA* sex chromosomal aneuploidy, *TP* true positive, *FP* false postive, *FN* false negative, *NPV* negative predictive value

**Fig. 1:**
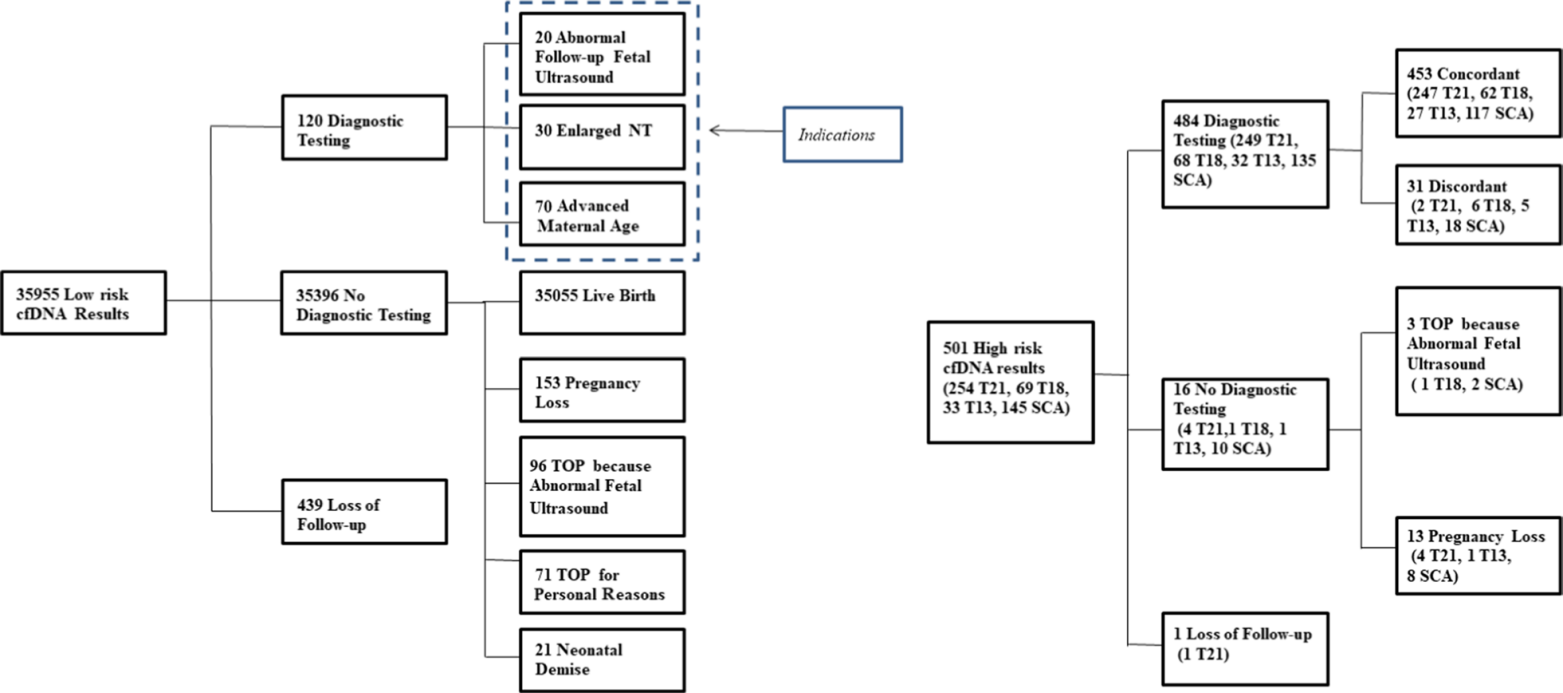
Outcomes of pregnancies with high-risk and low-risk cfDNA screening results. TOP, termination of pregnancy; NT, nuchal translucency

Follow-up data were available in 472/489 (96.5%) of women with high-risk results and singleton pregnancies, and 12/12 (100%) of women with high-risk results and twin pregnancies. In the cohort with low-risk NIPT results we received the results of invasive testing for 120 women (0.3%). Among these 120 cases none had T21, T18, or T13. In the 35,396 cases with normal NIPT results 153 experienced a pregnancy loss, 96 women opted for termination of pregnancy (TOP) because of fetal abnormalities at ultrasound, 71 chose TOP for personal reasons, and 21 had neonatal demise or other rare diseases (Fig. 1). No false negative results were reported to the laboratory in the 35,055 live births with normal NIPT results.

Confirmatory testing results were available in 484/501 (96.6%) of singleton and twin pregnancies with high-risk NIPT results (Fig. 1): 410 amniocentesis, 69 chorionic villus sampling, and 5 cases with products of conception after termination/miscarriage. The 17 cases without confirmatory test results were all singleton pregnancies: in 16 cases, intrauterine fetal death (IUFD) occurred or the pregnancy was terminated because of ultrasound anomalies without confirmatory testing, and in one case, no follow-up was available. In total, 448/479 high-risk NIPT results were concordant with karyotype or SNP array in chorionic villi or amniotic fluid cells, and 5/5 were concordant in products of conception after termination/miscarriage. There were 31/484 (0.08%) false positives: 2 T21, 6 T18, 4 T13 and 18 SCA in singleton pregnancies and 1 T13 in a twin pregnancy. Ten were ascertained in chorionic villi and 21 in amniotic fluid cells.

In total, 800 twin pregnancies and 6 triplet pregnancies were tested (Table 4). Confirmatory testing was performed by amniocentesis in all cases with abnormal NIPT results. Twelve dichorionic twin pregnancies were reported to be at high risk of being affected: 8 T21, 2 T18 and 1 T13. The T21 and T18 high-risk results were confirmed by follow-up testing, and the T13 was a false positive NIPT result. Of the six triplet pregnancies, one was reported as high risk for T21 by NIPT, which was confirmed in the amniotic fluid. No false negative cases were reported among the multiple pregnancies.

**Table 4:** NIPT performance for detecting trisomies 21, 18, 13 and sex chromosome aneuploidies in 800 twin and 6 triplet pregnancies

Twins n = 800	TP	FP	TN	Reported FN	Sensitivity	Specificity	PPV	NPV
					TP/(TP + FN)	TN/(TN + FP)	TP/(TP + FP)	TN/(TN + FN)
					%(95% Cl)	%(95% Cl)	% (95% Cl)	% (95% Cl)
Trisomy 21 (8)	8	0	792	0	100 8/8	100 792/792	100 8/8	100 792/792
					(59.7,100.0)	(99.39, 100.0)	(59.7, 100.0)	(99.39, 100.0)
Trisomy 18 (2)	2	0	798	0	100 2/2	100 798/798	100 2/2	100 798/798
					(19.79, 100)	(99.40, 100)	(19.78, 100.0)	(99.40, 100.0)
Trisomy 13 (1)	0	1	799	0		99.77 799/800		100 799/799
						(99.13, 100.0)		(99.99, 100.0)
Triplets n = 6								
Trisomy 21 (1)	1	0	0	0				

*CI* confidence interval, *TP* true positive, *FP* false positive, *FN* false negative

In 1807 assisted reproductive technology (ART) pregnancies, including 403 twin pregnancies and 16 egg donation pregnancies (Table 5), the test's performance showed 100% sensitivity and specificity for T21 and T18 and a slightly lower specificity for T13 (99.94%) and SCA (99.83%).

**Table 5:** NIPT Performance for Detecting Trisomies 21, 18, 13 and Sex Chromosome Aneuploidies in ART Pregnancies

ART pregnancies n = 1807	TP	FP	TN	Reported FN	Sensitivity	Specificity	PPV	NPV
					TP/(TP + FN)	TN/(TN + FP)	TP/(TP + FP)	TN/(TN + FN)
					%(95% Cl)	%(95% Cl)	%(95% Cl)	%(95% Cl)
T21	9	0	1798	0	100 9/9	100 1798/1798	100 9/9	100 1798/1798
					(62.88,100.0)	(99.73, 100.0)	(62.88, 100.0)	(99.79, 100.0)
T18	2	0	1805	0	100 2/2	100 1805/1805	100 2/2	100 1805/1805
					(19.79,100.0)	(99.73, 100.0)	(19.78, 100.0)	(99.73, 100.0)
T13	0	1	1806	0		99.94 1806/1807	–	100 1806/1806
						(99.51, 99.98)		(99.99, 100.0)
SCA	7	3	1797	0	100 7/7	99.83 1797/1800	70 7/10	100 1797/1797
					(64.57,100.0)	(99.23, 99.91)	(41.58, 98.42)	(99.99, 100.0)

*CI* confidenceinterval, *ART* assisted reproductive technology, *T21* trisomy 21, *T18* trisomy 18, *T13* trisomy 13, *TP* true positive, *FP* false positive, *FN* false negative, *PPV* positive predictive value

Of the 254 cases reported as high-risk for T21, 5 singleton pregnancies did not have confirmatory testing. Four of these cases ended in intrauterine fetal death (IUFD), and in one case, we did not have additional data. In 247/249 (97.2%) cases with confirmatory testing, T21 was confirmed, and 2 were false positives. This led to a specificity of 99.99% (95% CI 99.98, 100.0) and a PPV of 99.2 (95% CI 98.1, 99.3). If we were to consider the 5 cases without confirmatory testing as false positives, which is unlikely for the four cases ending in IUFD since T21 is known to be associated with a high risk of IUFD, the PPV for T21 for the entire cohort would drop to 97.2% (247/254). The PPV for T21 for the multiple pregnancy cohort was 100% (CI 59.7, 100.0) (9/9), and there were no positive cases without follow-up data. There were no reported discordant negative cases, resulting in a sensitivity of 100% (95% CI 98.47, 100.0) and NPV of 100% (95% CI 99.99, 100.0) (Tables 2, 3 and Additional file 1: Table S1).

Of the 69 cases reported as high-risk for T18, 68 had confirmatory results: 62/68 (91.3%) were true positives and 6 were false positives. One singleton pregnancy was terminated because of ultrasound anomalies without follow-up testing. This led to a specificity of 99.98% (95% CI 99.96, 100) and a PPV of 91.2% (95% CI 84.54, 97.86) (62/68). If we considered the case without confirmatory testing a false positive, which is unlikely in view of the ultrasound anomalies, the PPV would drop to 89.8% (62/69).The number of T18 cases in the multiple pregnancy cohort was too low (2) to calculate a PPV. No false negative T18 cases were reported. The T18 sensitivity was 100% (95% CI 94.17, 100.0) and the NPV was 100% (95% CI 99.99, 100.0) (Tables 2, 3 and Additional file 1: Table S1).

Of the 33 cases reported as high-risk for T13, 27 were confirmed by diagnostic testing, one ended in fetal demise without a confirmatory test, and 5 were false positives, resulting in a specificity of 99.99% (95% CI 99.97, 100.0) and a PPV of 84.4% (95% CI 83.15, 96.90) (27/32). If we were to consider the case without confirmatory testing a false positive, which is unlikely since T13 is known to be associated with a high risk of IUFD, the PPV would drop to 81.8% (27/33). The number of T13 cases in the multiple pregnancy cohort was too low (1) to calculate a PPV. No false negative T13 cases were reported. Thus, the T13 sensitivity was 100% (95%CI 87.54, 100.0), with an NPV of 100% (95% CI 99.99, 100) (Tables 2, 3 and Additional file 1: S1). In four out of the twelve false positive results for classic trisomies, a vanishing twin was identified (Additional file 1: Table S1).

NIPT indicated 145 singleton pregnancies with SCA. Eight 45,X pregnancies ended in fetal demise without confirmatory testing, and 2 pregnancies were terminated without confirmation (Table 2). A cytogenetic analysis of chorionic villi or amniotic fluid was performed in 135 cases and confirmed 117 positive cases; 18 cases were false-positive. This resulted in an overall specificity of 99.95% (95% CI 99.92, 99.97) and a PPV of 87.7% (95% CI 81.0, 92.38). The specificity was lowest for 45,X and highest for 47,XYY. No false negatives were reported, but this was not expected, since, with the exception of some cases of Turner syndrome, newborns with an SCA do not have a phenotype that prompts karyotyping. Therefore, we did not tabulate the sensitivity or NPV (Table 6). The 145 SCAs included 69 cases of monosomy X, which was 13.8% of the total number of abnormal results. Fifty-two out of these 69 monosomy X cases were confirmed at follow-up karyotyping, 9 were false positives, and 8 cases ended with IUFD without cytogenetic confirmation. Two of the 52 confirmed cases were mosaic in the foetus: 1 case of 45,X[68]/46,X,i(X)(q10)[32] and 1 case of 45X/46XY. One of the 52 confirmed cases was a de novo partial deletion: 46,X,del(X)(q?). NIPT indicated 24 cases of 47,XXX, which was 4.79% of the abnormal results, with 20/24 cases confirmed at follow-up karyotyping and 4 false positives. NIPT indicated 42 cases with 47,XXY, 8.38% of the abnormal results, with 35 of those 42 being confirmed at follow-up karyotyping and 5 false positives. Two had TOP before the gestational age of 90 days, the legal gestational age for termination of pregnancy for social reasons in Italy [23], and remained without cytogenetic confirmation. In the cases where NIPT indicated 47,XXY, karyotyping in amniotic fluid identified one case with 48,XXYY and one case with 49,XXXXY. NIPT also indicated 10 cases with 47,XYY, 1.99% of the abnormal results, and all were confirmed by follow-up karyotyping (Table 6). Finally, we identified 11 cases of maternal sex chromosome aneuploidy. Maternal karyotyping was recommended because of high LLR and/or NCV_X scores: 6 of these were mosaic for 45,X0, 4 were identified as 47,XXX, and one was a mosaic 47,XXX/45,X0 (Additional file 2: Table S2). None of these women had conceived naturally. Overall, 29% of the abnormal results were anomalies of the sex chromosomes; 51% were trisomy 21 and 20% were trisomy 18 or 13.

**Table 6:** NIPT Performance for detecting individual sex chromosome aneuploidies

n = 145	TP	FP	TN	Reported FN	Specificity TN/(TN + FP) %(95% Cl)	PPV TP/(TP + FP) %(95% Cl)
47,XXX	20	4	111	0	96.52 111/115	83.3 20/24
					(93.12,98.92)	(68.40, 98.20)
47,XXY°	35	5	95	0	95.00 95/100	87.5 35/40
					(89.62, 98.01)	(78.30, 97.90)
45, X°°	52	9	74	0	89.16 74/83	85.2 52/61
					(81.09, 94.33)	(79.16,94.84)
47,XYY	10	0	125	0	100 125/125	100 10/10
					(96.28, 100)	(96.28, 100.0)

*CI* confidence interval, *TP* true positive, *FP* false positive, *FN* false negative

°Karyotyping in amniotic fluid revealed one was a case of 48, XXYY and one was a case of 49, XXXXY

°°Karyotyping in amniotic fluid revealed one partial deletion 46,X,del(X)(q?)*dn* and two mosaics: 45X/46XY and mos45,X[68]/46,X,i(X)(q10)[32]

### Test failure

**Fig. 2:**
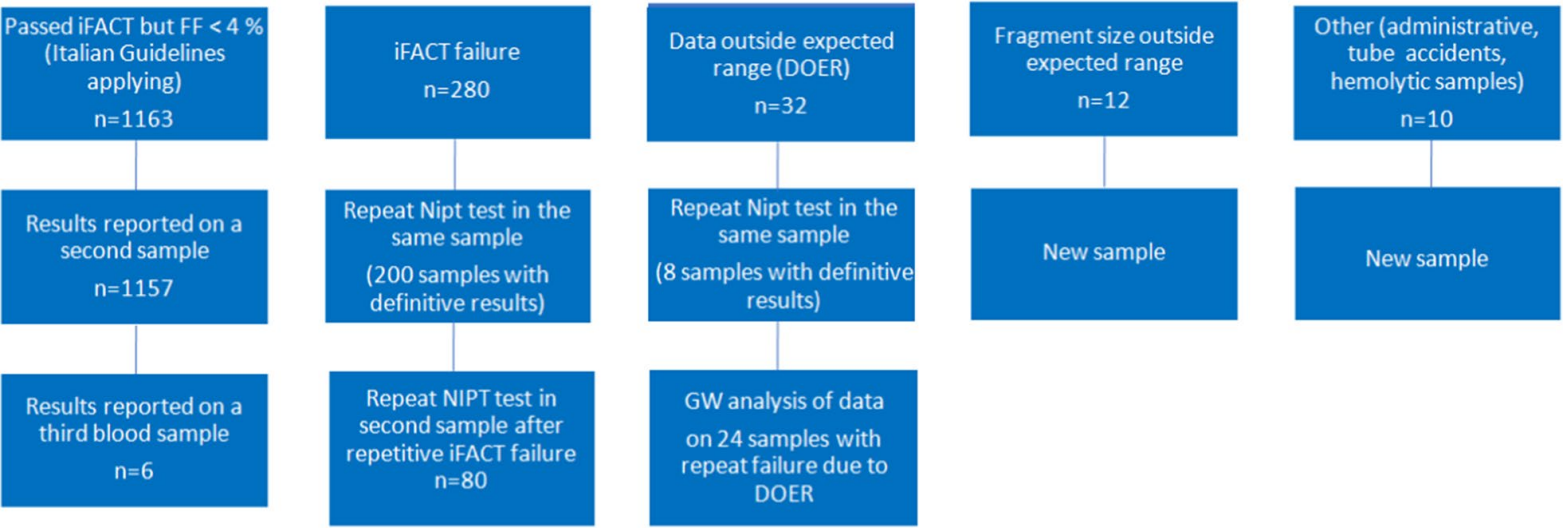
Test failures (n = 1497) and decision tree for samples that failed at the first attempt

**Fig. 3:**
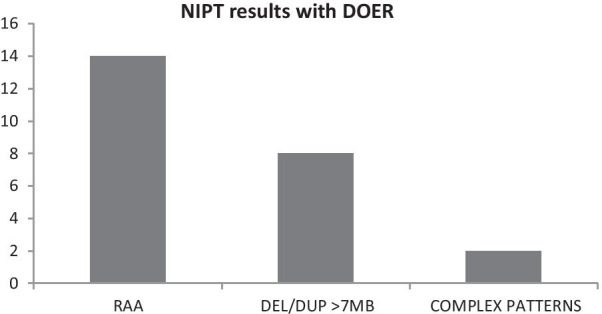
Results of the secondary genome-wide analysis in 24 samples with repetitive data outside of the expected range (DOER) in the first blood sample. RAA, rare autosomal aneuploidy; DEL/DUP, deletions/duplications; COMPLEX PATTERNS, complex maternal genomic profiles

In 1497 (4.1%) of the 36,456 samples, the results could not be reported at the first attempt (Fig. 2). In 10 (0.03%) cases, there were “administrative errors”, including problems with collection or transportation and haemolytic samples. In 1163 (3.2%) cases, the fetal fraction was below 4% and failed according to Italian regulations even though they had passed iFACT [24, 25]. An additional 280 cases failed iFACT; 32 samples classified as data outside of the expected range (DOER) and 12 samples failed because of fragment size out of the expected range. The test was repeated on a new sample in 1163 women with FF < 4%, and in 6 of these women, the test had to be repeated twice before a result could be issued. In all women with initial failure because of FF < 4%, a result could be reported in a new sample where the FF eventually surpassed 4%. The 280 samples with iFACT failure and the 32 with DOER were repeated on the second tube drawn at the first sampling and stored; of these, 208 were reportable. A second blood sample was taken from 80 patients with a second iFACT failure, 12 patients with failure due to fragment size outside of the expected range and 10 patients with “administrative” reasons for primary failure. In 24/32 samples with DOER that could not be reported after a second tube testing, the data of all autosomes were analysed; rare autosomal trisomies, large deletions and/or duplications (> 7 MB), or complex maternal genomic profiles were seen in all 24 samples (Fig. 3). For 1443 out of 1453 repeated tests, a conclusive result could ultimately be issued. None of the failures affected twin or triplet pregnancies. In total, including repeated testing, a conclusive result was reported in 99.97% of singleton pregnancies and in all multiple pregnancies.

## Discussion

We report on a large cohort of consecutive pregnancies referred to our laboratory for WGS-based NIPT for classic trisomies and SCA in singleton pregnancies and classic trisomies in twin and triplet pregnancies. Follow-up data were available in 96.6% of pregnancies with abnormal NIPT results. The results showed that test performance in singleton and twin pregnancies was equal and test failures were uniformly low for both cohorts. WGS-based NIPT can safely be offered to women who are pregnant with multiple foetuses. This is relevant, since in addition to the risk of an abnormal result, the risk of invasive test procedures is higher in twin pregnancies. The WGS-based NIPT used has the ability to estimate fetal fraction based on read lengths and coverage profiles, the ability to take into account the effect of aneuploidies in non-targeted chromosomes on aneuploidy scoring, and the ability to compare aneuploidy scoring with fetal fraction estimates. These features and adherence to a strict decision tree for samples that failed at the primary attempt allowed a high test accuracy and a very low overall non reporting rate for both singleton and multiple pregnancies. Cell-free DNA-based NIPT has revolutionized prenatal screening for chromosome anomalies and, to date, is the best-performing screening method for common autosomal aneuploidies, especially trisomy 21, in elevated-risk and low-risk patients [26–29]. The primary strengths of the present study are the robust follow-up data and the inclusion of a considerable group of (affected) multiple pregnancies. The overall sensitivity of NIPT in our cohort was 100% for T21, T18, and T13, and the specificity was 99.99% for T21, 99.98% for T18, and 99.99% for T13. The PPVs were 99.2% for T21, 91.2% for T18, and 84.4% for T13, which dropped to 97.2%, 89.8% and 81.8% if all cases with high-risk NIPT results but without follow-up data were considered false positives. With regard to SCAs, only tested for singleton pregnancies, the sensitivity and specificity were 100% and 99.95%, respectively. For SCA, one must keep in mind that due to the lack of a newborn phenotype, the sensitivity and specificity of prenatal tests for SCA will always be an overestimate. The test performance for the three classic trisomies in our cohort was comparable to previous large, real-life cohorts with lower follow-up percentages [30, 31].

A limitation of this study is that, as in many studies [14], cytogenetic information was not available on most pregnancies ending with fetal loss or miscarriage, terminated because of ultrasound anomalies, or ending in newborn demise. Here, there were 270 such cases (Fig. 1). Although in the NIPT cases with an abnormal result, the adverse pregnancy outcomes are most likely related to the anomaly identified with NIPT, in the NIPT cases with normal results, there might be false negatives amongst the pregnancy loss cases and cases may have remained unreported. This means that the sensitivity may have been overestimated. False-negative (FN) cfDNA screening cases have a high clinical impact on patients and clinicians. The main reasons for discordant results between cfDNA screening and fetal karyotype are low fetal fraction (FF) and mosaicism involving the placenta or the foetus to different extents [32–36]. In our study, we were not confronted with any false negative results. Our technology derives statistical scores (LLR scores) for each autosome of interest by comparing the coverage regions that can be affected by aneuploidy with a set of reference chromosomes. The LLR score is computed for each sample and takes into account the coverage and estimated fetal fraction. LLR scores reflect the trisomic fraction relative to the fetal fraction, and equivocal results are followed up with either retesting or the recommendation to have an invasive prenatal test. Cases that did not meet the FF thresholds were resampled.

Given the rarity of twins affected by fetal trisomy, data substantiating the performance of cfDNA-based testing in twin pregnancies are limited on the one hand, but badly needed on the other hand because the risks of invasive testing are higher in twin pregnancies [37]. The largest cohort on NIPT in multifetal gestations was published by Dyr et al. [38] in 2019. This retrospective study included 23,986 twin and 709 triplet pregnancies. The average nonreportable rate was 5.95%, with 6.05% in twin pregnancies and 21.3% in triplets, mostly because of low fetal fraction. Follow-up was limited to 50 cases. Performance was calculated assuming that all FPs and FNs had been reported, leading to sensitivities of 98.4%, 97.16% and > 99.99% for T21, 18, and 13, respectively, and PPVs of 99.08%, 99.28%, and 88.71%, respectively. In the current study, we analysed NIPT performance in 800 twin pregnancies. We had a 100% follow-up of all cases with abnormal NIPT results and assumed that false negatives would be reported. The sensitivity and specificity for T21 were 100% (95% CI 56.55, 100.0) and 100% (95% CI 99.13, 100.0), respectively. We found one false positive result for T13 in a dichorionic IVF pregnancy, in which a discrepancy between cfDNA and fetal karyotype was demonstrated in amniotic fluid cells. There was an insufficient number of T18 and T13 twin cases to calculate performance metrics.

A recent systematic review of 7 studies with 1141 twin pregnancies indicated a similar performance of NIPT for the detection of T21 in singleton and twin pregnancies [15]. The authors stated that NIPT for T21 performed substantially better than a first-trimester combined test or second-trimester biochemical test and should therefore be preferred. In France, all women with twin- or higher-order multiple pregnancies have been given access to reimbursement for NIPT for T21 (http://www.cngof.net/Partenaires/JO/joe-2018-12-20-depistage-T21.pdf). For trisomies 18 and 13, the number of cases in the literature is too small for an accurate performance assessment [15].

All professional societies, including the Italian Society of Human Genetics (SIGU), characterize cfDNA as a screening method and recommend that cases with high-risk cfDNA results receive genetic counselling and be offered an invasive prenatal diagnosis for confirmation [23, 24, 39, 40]. In our cohort, 84.7% (410/484) of high-risk cases had amniocentesis and 14.3% (69/484) had chorionic villus sampling. This choice in favour of amniocentesis reflects the advice given at post-test counselling in view of the possibility of false positive or mosaic results of confirmatory testing in chorionic villi [10, 11, 32]. Only 3 out of 501 patients with high-risk NIPT results (0.6%) declined the offer of an invasive prenatal diagnosis and terminated their pregnancies without confirmatory testing because of structural abnormalities revealed by ultrasound. In two of these terminations NIPT reported a SCA, and the fetuses showed unilateral hydronephrosis. One hundred and twenty (0.3%) patients with low-risk NIPT results opted for invasive prenatal diagnosis, and most of them had risk factors for aneuploidy, such as enlarged nuchal translucency or advanced maternal age.

Non reportable rates after redrawing differ per technology, and failure rates up to 3.89% have been described [41]. Reasons include a low fetal fraction (FF), multiple gestations, mosaicism, alterations in the maternal genome and aneuploidies in nontargeted comparator chromosomes. Our differentiated decision tree for handling primary failures resulted in a very low final rate of nonreportable results (10/36,456 = 3 per 10.000 samples). Of particular interest are the 24 samples that failed at the first attempt because of data outside of the expected range (DOER) and failed again for the same reason at retesting of a second tube obtained during the first blood draw. In each of these samples, genome-wide analysis revealed either a rare autosomal aneuploidy, large (> 7 MB) deletion or duplication, or a complex maternal genomic profile in nontargeted chromosomes (Fig. 3). It has been reported that with targeted NIPT technologies, test failures occur in case of trisomies in autosomes other than chromosomes 21, 18 or 13 [42]. The classification of non-reportable results is important for the choice between retesting using the same sample, retesting using a new sample, recommending invasive testing, or performing a genome-wide analysis of all autosomes [43]. The latter is only possible if NGS-based technologies are used.

Finally, an analysis of the NCV_X, NCV_Y and LLR scores of the sex chromosomes upon identification of false positive cases for SCA allowed us to identify 11 maternal (mosaic) sex chromosomal aneuploidies, including 4 cases of 47,XXX, 6 cases of mosaic Turner and one case of mosaic 47,XXX/46,XX/45,X. None of these women had conceived spontaneously. This information will allow personalized counselling and care for (pregnant) women with mosaic Turner syndrome, who have an increased risk of cardiovascular anomalies and premature ovarian failure [44, 45], as well as advising against NIPT for sex chromosomes in future pregnancies. Other studies have reported maternal SCA as the basis for discordance between NIPT results and fetal karyotype [10, 11, 46–48]. Pregnant women known to have an SCA should be counselled that NIPT for sex chromosomes does not provide reliable results, in this situation.

## Conclusion

We have presented real-life data showing high test accuracy for a WGS-based cfDNA non-invasive prenatal test that is applicable to both singleton and multifetal pregnancies. Stringent application of a decision tree for failed samples resulted in a non-reportable rate of 3 in 10,000 samples in singletons and no non-reportable cases in multiple pregnancies.

## Supplementary Information

**Additional file 1**. **Table S1.** Overview of false positive NIPT results for trisomies 21, 18, and 13. **Table S2.** Overview of Maternal Sex Chromosome Aneuploidies suspected in NIPT results because of LLR score indicative of maternal aneuploidy.

## Data Availability

All data generated or analysed during this study are included in this published article (and its supplementary information files). Protocols and deidentified, aggregated data that underlie the results reported in this article are available for non-commercial scientific purposes upon reasonable request from the corresponding author. For privacy reasons raw data are not publicly available.

## Abbreviations

AC: Amniocentesis
CVS: Chorionic villus sampling
cfDNA: Circulating cell-free DNA
MPS: Massively parallel sequencing
NIPT: Non-invasive prenatal test
PPV: Positive predictive value
SCAs: Sex aneuploidies
WGS: Whole-genome sequencing
ART: Assisted reproductive techniques
TOP: Termination of pregnancy
FF: Fetal fraction
