# Supplementary material for: Performance of cell-free DNA sequencing-based non-invasive prenatal testing: experience on 36,456 singleton and multiple pregnancies
Source: BMC Med Genomics. 2021 Mar 30;14:93. doi: 10.1186/s12920-021-00941-y (PMC8011149; doi:10.1186/s12920-021-00941-y)
Supplement: Supplementary file 1 — Additional file 1. Table S1. Overview of false positive NIPT results for trisomies 21, 18, and 13. Table S2. Overview of Maternal Sex Chromosome Aneuploidies suspected in NIPT results because of LLR score indicative of maternal aneuploidy. [file 12920_2021_941_MOESM1_ESM.docx]

**Table S1**: Overview of false positive NIPT results for Trisomies 21, 18, and 13.

| **NIPT result** | **MA (y)** | **GA at NIPT (w)** | **Other information** | **Prenatal diagnostic test result** | **Pregnancy outcome** | **Postnatal diagnostic test result** |
| --- | --- | --- | --- | --- | --- | --- |
| T21 | ≤35 | 16+3 |  | AC: normal | Live born female, no congenital anomalies | Placenta: normal karyotype |
| T21 Borderline LLR score (7.47),FF=4%) | ≥35 | 14+2 | Presence of a vanishing twin  (Retesting at 16+2 did not indicate T21) | AC: normal | Live born male, no congenital anomalies | Placenta: normal karyotype |
| T18 | ≥35 | 15+2 |  | AC: normal | Live born female, no congenital anomalies | Placenta: normal karyotype |
| T18  Borderline LLR score (77.14), FF=14% | ≤35 | 12+3 | Presence of a vanishing twin  (Repetition at 16+3 again showed a borderline value for T18) | AC: normal | Live born male, no congenital anomalies | Placenta: normal karyotype |
| T13, T18 | ≥35 | 11+2 |  | AC: normal | Live born female, no congenital anomalies | Placenta: normal karyotype |
| T18,  Borderline LLR score (36.89,  FF=7%) | ≥35 | 12+3 | Presence of a vanishing twin | AC: normal | Live born male, no congenital anomalies | Placenta: normal karyotype |
| T18 | ≥35 | 13+6 |  | AC: normal |  |  |
| T18 | ≥35 | 15+3 |  | AC: normal |  |  |
| T13  Borderline  LLR score (26.148),  FF=6% | ≥35 | 10+6 | Presence of a vanishing twin | AC: normal | Live born female, no congenital anomalies |  |
| T13 twin pregnancy | ≥35 | 14+1 |  | AC: normal in bothfetus | Live born female and male, no congenital anomalies |  |
| T13 | ≥35 | 13+1 |  | AC: normal | Live born female and male, no congenital anomalies |  |
| T13 | ≤35 | 11+2 |  | AC: normal | Live born female and male, no congenital anomalies |  |

T21, trisomy 21; T18, trisomy 18; T13, trisomy 13; FF, Fetal Fraction; AC Amniocentesis

**Table S2.** Overview of Maternal Sex Chromosome Aneuploidies suspected in NIPT results because of LLR score indicative of maternal aneuploidy.

| **Maternal Age (yrs)** | **Gestational Age** | **NIPT Result** | **Maternal karyotype** | **Type of Invasive Test** | **Result of Invasive Test Karyotype** |
| --- | --- | --- | --- | --- | --- |
| ≤35 | 20+1 | Normal for T21,18,13; failure for sex chromosomes; suspicion maternal 47,XXX | 47,XXX | AC | 46,XX |
| ≥35 | 10+2 | Normal for T21,18,13; failure for sex chromosomes; suspicion maternal 45,X mosaic | mos45,X[17]/46,XX[33] | AC | 46,XX |
| ≤35 | 10+6 | Normal for T21,18,13; failure for sex chromosomes; suspicion maternal 47,XXX | 47,XXX | AC | 46,XX |
| ≤35 | 11+2 | Normal for T21,18,13; failure for sex chromosomes; suspicion maternal 45,X mosaic | mos45,X[20]/46,XX[30] | AC | 46,XX |
| ≤35 | 15+3 | Normal for T21,18,13; failure for sex chromosomes; suspicion maternal 45,X mosaic | mos45,X[19]/46,XX[31] | AC | 46,XX |
| ≥35 | 11 | Normal for T21,18,13; failure for sex chromosomes; suspicion maternal 45,X mosaic | mos45,X[8]/46,XX[42] | AC | 46,XX |
| ≥35 | 12+2 | Trisomy for T18; failure for sex chromosomes; suspicion maternal 45,X mosaic | mos45,X[30]/46,XX[20] | AC | 47,XY+18 |
| ≤35 | 10+2 | Normal for T21,18,13; failure for sex chromosomes; suspicion maternal 47,XXX | 47,XXX[89]/45,X[7]/46,XX[4] | AC | 46,XX |
| ≤35 | 11+3 | Normal for T21,18,13; failure for sex chromosomes; suspicion maternal 47,XXX | 47,XXX | AC | 46,XX |
| ≥35 | 11 | Normal for T21,18,13; failure for sex chromosomes; suspicion maternal 45,X mosaic | mos45,X[6]/46,XX[44] | AC | 46,XX |
| ≤35 | 12 | Normal for T21,18,13; failure for sex chromosomes; suspicion maternal 47,XXX | 47,XXX | AC | 46,XX |

T21, trisomy 21; T18, trisomy 18; T13 trisomy 13; AC Amniocentesis
